# Dirac Electrons with Molecular Relaxation Time at Electrochemical Interface between Graphene and Water

**DOI:** 10.3390/ijms251810083

**Published:** 2024-09-19

**Authors:** Alexey V. Butko, Vladimir Y. Butko, Yurii A. Kumzerov

**Affiliations:** Ioffe Institute, Polytechnicheskaya 26, 194021 St. Petersburg, Russia

**Keywords:** graphene, molecule, nanostructure, water, quantum capacitance, Dirac cone, impedance spectroscopy, sensor, relaxation time, interfacial bonding

## Abstract

The time dynamics of charge accumulation at the electrochemical interface between graphene and water is important for supercapacitors, batteries, and chemical and biological sensors. By using impedance spectroscopy, we have found that measured capacitance (C_m_) at this interface with the gate voltage V_gate_ ≈ 0.1 V follows approximate laws C_m_~T^1.2^ and C_m_~T^0.11^ (T is V_gate_ period) in frequency ranges (1000–50,000) Hz and (0.02–300) Hz, respectively. In the first range, this dependence demonstrates that the interfacial capacitance (C_int_) is only partially charged during the charging period. The observed weaker frequency dependence of the measured capacitance (C_m_) at frequencies below 300 Hz is primarily determined by the molecular relaxation of the double-layer capacitance (C_dl_) and by the graphene quantum capacitance (C_q_), and it also implies that C_int_ is mostly charged. We have also found a voltage dependence of C_m_ below 10 Hz, which is likely related to the voltage dependence of C_q_. The observation of this effect only at low frequencies indicates that C_q_ relaxation time is much longer than is typical for electron processes, probably due to Dirac cone reconstruction from graphene electrons with increased effective mass as a result of their quasichemical bonding with interfacial molecular charges.

## 1. Introduction

**Figure 1:**
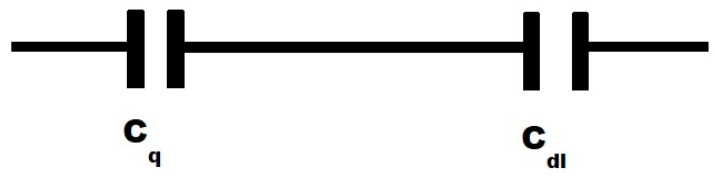
Schematic illustrations of equivalent circuits of C_int_.

Graphene-based nanostructures interfaced with aqueous and other electrolytic solutions are promising candidates for the fabrication of a new generation of various devices. Examples of these devices include supercapacitors [1,2,3,4,5,6], batteries [7,8,9,10,11], and chemical and biological sensors [12,13,14,15,16,17,18,19,20,21,22,23,24,25,26,27,28,29]. The time dynamic of charge accumulation at these interfaces is important because this dynamic strongly affects these applications. Understanding this dynamic requires impedance studies in a broad range of frequencies, including a low frequency range. However, the reported impedance measurements at these interfaces were mainly fulfilled at frequencies in the range of 5–1000 Hz [12,30], and low frequency measurements that are required for obtaining the electrostatic parameters of these interfaces are very limited [3,31]. Moreover, experimental studies by impedance spectroscopy were mainly reported at the graphene interface with solutions of various salts; meanwhile, practically important interfaces between graphene and water are less studied experimentally. The capacitance of the electrochemical interface (C_int_) between graphene and water was described as being connected in series C_dl_ and C_q_ (see Figure 1) by a number of researchers [12,30,32,33,34,35,36].

These capacitances are considered to be important parameters and are currently under intensive investigation [3,30]. Quantum capacitance that was introduced for the first time in work [37] is primarily important for low-density-of-states systems, such as graphene. The quantum capacitance of graphene is directly related to the density of states of Dirac fermions that form Dirac cones in which the electronic energy and momentum have a linear dispersion [38]. The two conical surfaces for electrons and holes touch each other at the Dirac point and form a zero-band gap semimetal [38]. In spite of the importance of C_q_ and C_dl_, the time dynamics of these parameters remain largely unexplored [39]. Therefore, in this work, we focus on obtaining the frequency dependence of the electrical capacitance at the interfaces of graphene with deionized water in a broad frequency range from 0.02 Hz to 50 kHz, as well as on establishing the main dynamical features of Dirac electrons in these systems.

## 2. Results and Discussion

**Figure 2:**
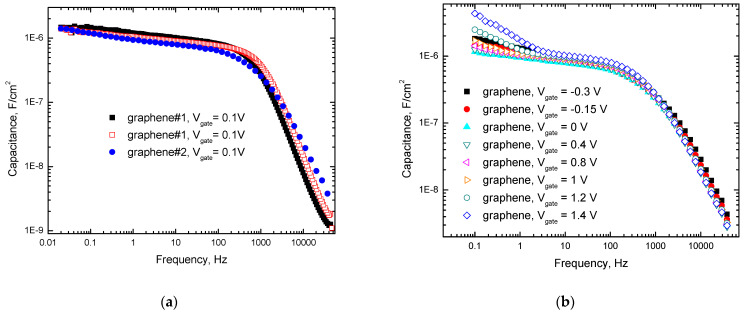

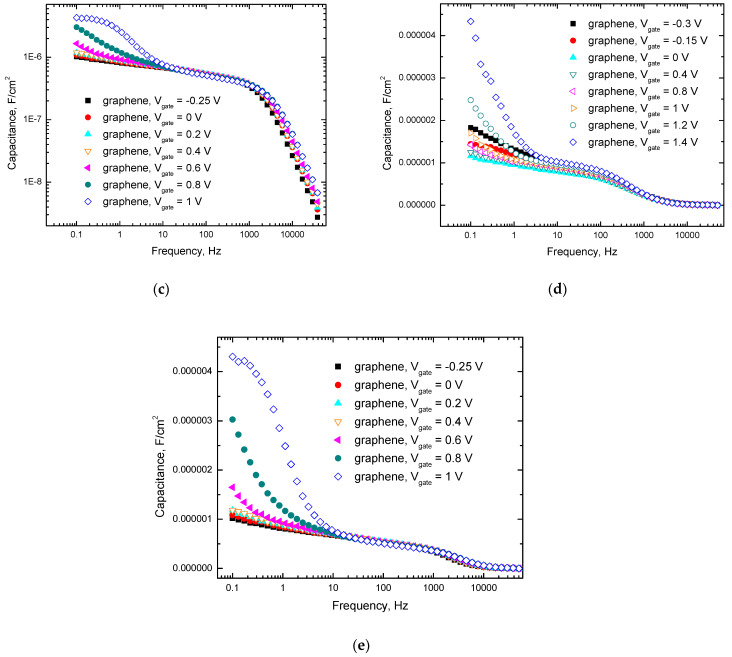
Typical frequency dependencies of the capacitance measured in water between graphene and gate electrodes normalized to the graphene surface area (**a**) at V_gate_ = 0.1 V with a single gold wire gate electrode (graphene#1) and a gate electrode of 6 gold wires (graphene#2) on the logarithmic scale, (**b**) at 8 different gate voltages with a gate electrode of 6 gold wires on the logarithmic scale, (**c**) at 7 different gate voltages with a single gold wire gate electrode on the logarithmic scale, (**d**) at 8 different gate voltages with a gate electrode of 6 gold wires on the linear scale, and (**e**) at 7 different gate voltages with a single gold wire gate electrode on the linear scale.

Figure 2a–e shows the typical capacitances (C_m_) measured at different gate voltages between graphene and two different types of gate electrodes in water.

One can see from Figure 2a–c that the measured capacitance (C_m_) at this interface with the applied gate voltage V_gate_ ≈ 0.1 V follows approximate laws C_m_~T^1.2^ and C_m_~T^0.11^ (T is V_gate_ period) in frequency ranges (1000–50,000) Hz and (0.02–300) Hz, respectively. Figure 2a also demonstrates the stability of the studied interfaces over time and that the observed effects are reproducible.

**Figure 3:**
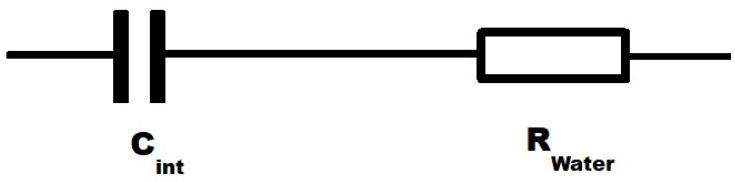
Schematic illustrations of equivalent circuits of C_m_.

To explain the observed effects, we propose a charge accumulation model at the graphene interface with water. In this model, C_m_ in frequency ranges (1000–50,000) Hz is mainly limited by the charge passing through the water during the charging period under the applied gate voltage, and C_m_ is only a fraction of the electrostatic value of the interfacial capacitance (C_int_). The observed weak superlinear dependence of C_m_ on T in this frequency range indicates that molecular charges participate in accelerated motion under the applied voltage. Taking into account that C_int_ is connected in series with the resistance of water in the measuring circuit (see Figure 3), this dependence implies that C_m_ is mainly limited by the amount of charge passing through the resistance of water during the charging period.

The observed weaker (than in the previously discussed frequency range) dependence at frequencies below 300 Hz can be explained by assuming that, in this range, T is long enough to charge C_int_, C_m_ is primarily determined by the double-layer capacitance (C_dl_) and the graphene quantum capacitance (C_q_), and the molecular relaxation of these capacitances plays an important role in the frequency dependence of C_m_.

**Figure 4:**
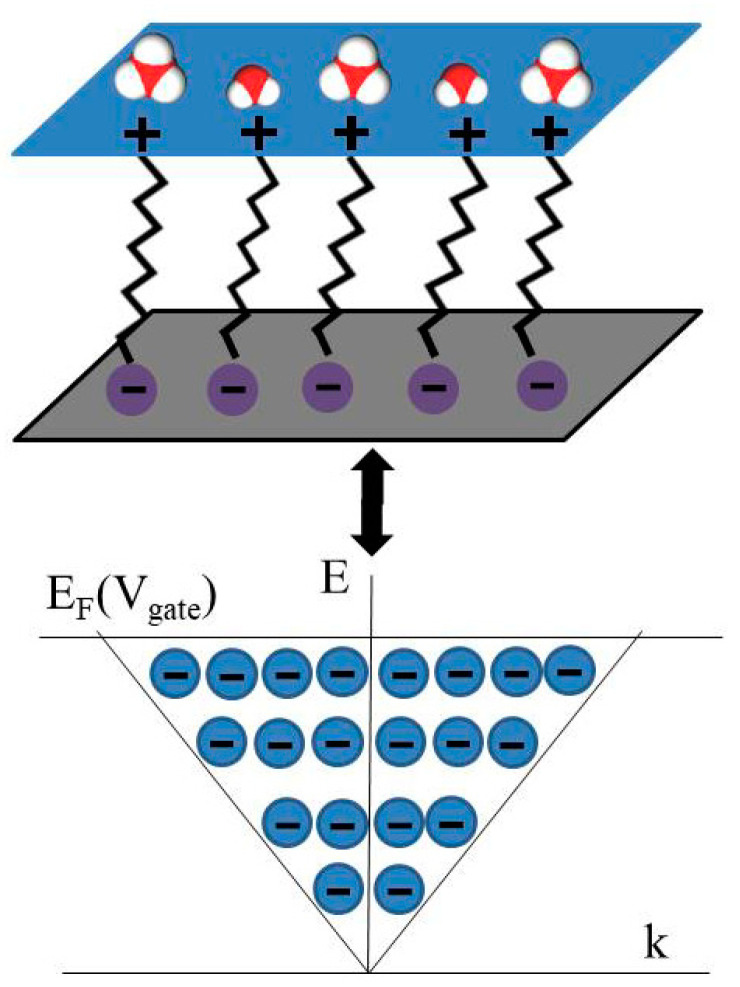
Schematic illustrations of the bonding between Dirac electrons in graphene and molecular charges at the electrochemical interface between graphene and water and of the Dirac cone formed from the bonded electrons.

One can also see from Figure 2b–e that C_m_ has a voltage dependence approximately below 10 Hz. This dependence is likely related to the voltage dependence of C_q_ that is given by the formula [30] C_q_ ≈ (2 e^3^ V_ch_)/(π ћ^2^ ν_F_^2^), where ћ is the Planck constant, e is an electron charge, ν_F_ ≈ c/300 is the Fermi velocity of the Dirac electrons, and V_ch_ is the potential of graphene. It was recently suggested [40] that electrochemical impedance spectroscopy measurements of the chemical capacitance in contact with electrolytes enable the direct analysis of the density of states (DOS) properties. Experimentally, Dirac cone manifestations in capacitance measurements at the electrochemical interfaces with graphene were observed previously [3,30]. In the vicinity of the Dirac point, graphene bares some similarities to the quantum metallic state studied [41,42] in quasi-2D metallic films. In particular, the maximal resistance of graphene is close to the quantum resistance level [38], and electron tunneling DOS suppression is linear [43]. This suppression likely plays a role in the limitation of the quantum capacitance from above, and the fundamental origin of the voltage dependence of C_q_ is the voltage-controlled Dirac cone reconstruction in graphene. The observation of the gate voltage dependence of C_m_ in this paper only at low frequencies indicates that C_q_ relaxation time is by orders of magnitude longer than is typical for electron processes. Defects and impurities in graphene samples can decrease the charge carrier mobility in graphene and increase the quantum capacitance relaxation time. However, this mechanism is limited due to the typical density of defects being as low as 5·10^10^ cm^−2^ [44] for these graphene samples. We primarily attributed the observed increase in the relaxation time of the quantum capacitance to quasichemical bonding between Dirac electrons in graphene and molecular charges at the interface (see Figure 4).

This Figure illustrates bonding between electrons in graphene and molecular charges at the electrochemical interface between graphene and water, and also shows the Dirac cone formed by the bonded electrons. As one can see from Figure 2b–e, the reconstruction of the Dirac cone by applying gate voltage requires a relaxation time that is greater than 0.1 s. This relaxation time is more typical for molecules rather than for electrons. Therefore, Dirac electrons at the interface between graphene and water are bonded to molecular charges and behave as quasiparticles with an effective mass that is similar to the mass of the water molecules.

Future studies are required to establish the applicability of the observed effects. Among these studies are investigations of graphene obtained by CVD and exfoliation methods with a surface roughness, a defect density, and electronic properties that may be different from the properties of graphene fabricated by the thermal decomposition of silicon carbide. Another interesting direction for future research is the investigation of the observed effects in bilayer or trilayer graphene that often demonstrate qualitatively different properties from the properties of monolayer graphene (see for instance [45]). The quantum capacitance of graphene and double-layer capacitance plays an important role at the electrochemical interfaces with carbon nanotubes (see, for example, [46]). For this reason, effects observed in our work may also be relevant at these interfaces. Our findings are important for understanding the charging and discharging process in supercapacitors and sensor applications. The speed dynamic of these processes can be controlled by choosing the types of charged molecules and their concentrations. The observed dependence of the measurable parameter (quantum capacitance) on bonding between electrons and molecular charges at the electrochemical interfaces opens additional opportunities for selective molecule detection in sensor applications.

## 3. Materials and Methods

In this work, we study the dynamics of capacitance at the electrochemical interface between graphene and water by low frequency impedance spectroscopy. We use monolayer graphene fabricated by the thermal decomposition of silicon carbide in work [15]. The growth method and the Raman structural characterization of graphene samples are thoroughly discussed in that paper [15]. We apply silver paste contacts to the graphene surface to prevent possible damage to graphene from the alternative lithographic process of electrode preparation. To prevent the degradation of the electrodes, we cover the silver paste contact pads with epoxy glue. The samples with the contacts would typically be subject to temperature treatment at 80–100 °C for up to 2 h in a dry box. The typical distance between electrodes on graphene, their length, and their typical width are between (1–1.5) mm, (3–4 mm), and (2.5–3) mm, respectively. To ensure that the contacts to graphene are ohmic [3], we compare 2 and 4 contact measurements and observe the approximate linear scaling of the graphene resistance with the length of the graphene sample. In this work, we use non-faradaic capacitors with blocking polarizable electrodes that carbon (graphene) and gold form at the interface with water [47]. Gold wires 50 µm in diameter are positioned ~200 µm away from graphene to act as gating electrodes. We study samples with gating electrodes made with a single gold wire and with six gold wires. Deionized water is positioned between the gating electrode and graphene for electrical impedance spectroscopy (EIS) measurements. Nonpolarizable reference electrodes such as Ag/AgCl were not used to avoid the addition of anions to the deionized water. Potentiostat-galvanostat Electrochemical Instruments P-45X, containing an FRA-24M module for measuring electrochemical impedance, is used for our measurements. This device allows electrochemical impedance measurements automatically calibrated in a broad frequency range. We check that this calibration is in good agreement with nominal capacitance values for a few commercially available capacitors and with results of a few fixed-frequency measurements, fulfilled by using AKIP 6108 LCR meter and Agilent U1242B multimeter (Santa Clara, CA, USA). All reported measurements have been made at the graphene interface with water at room temperature (≈20 °C), normal atmospheric pressure (≈760 mm Hg), and with an air humidity of about (≈70%). These parameters are within the certified spec range of P-45X. The input resistance of P-45X potentiostat-galvanostat is 10^12^ Ω. The impedance measurable range is from 5 mΩ up to 50 MΩ. The entire frequency range for P-45X potentiostat-galvanostat with the FRA-24M module is from 0.1 mHz to 1 MHz. In our experiments, a frequency range from 0.02 Hz to 50 kHz has been chosen to ensure that parasitic capacitances are negligible and that the measured parameters are in the measurable range according to the spec of the P-45X with the FRA-24M module. Impedance is measured between the gate electrode and graphene for different applied gate voltages ranging from −0.2 V to 1.4 V with DC offset scans within the amplitude range of ±20 mV. This asymmetric voltage range is chosen to minimize possible hysteretic phenomena and possible water decomposition [3,44]. Due to the mentioned blocking nature of the contacts between water and electrodes, the capacitance (C_m_) between the gate electrodes and graphene is obtained from the formula Z = 1/(i2πfC_m_), where f is the frequency of the applied gate voltage, i is the square root of negative 1, and Z is the measured impedance.

## 4. Conclusions

The frequency dependence of the electrical capacitance at the interfaces of graphene with deionized water has been studied in the frequency range from 0.02 Hz to 50 kHz. We have found that C_m_ at this interface with the gate voltage V_gate_ ≈ 0.1 V follows approximate laws C_m_~T^1.2^ and C_m_~T^0.11^ in frequency ranges (1000–50,000) Hz and (0.02–300) Hz, respectively. The observed dependence in the first frequency range is consistent with an assumption that C_m_ is only a fraction of the electrostatic value of C_int_ and is mainly determined by the accelerated motion of the molecular electrical charges in water to the graphene electrode under the applied gate voltage. Below 300 Hz, our model assumes that T is long enough to charge C_int_ and C_m_ is primarily determined by the double-layer capacitance (C_dl_) and the graphene quantum capacitance (C_q_). The observed frequency dependence of C_m_ in this range can be explained by the molecular relaxation of these capacitances. The obtained data on voltage dependence of C_m_ indicate that the gate voltage-dependent reconstruction of the Dirac cone requires a relaxation time that is greater than 0.1 s, which is much longer than is typical for electron processes, probably due to the quasichemical bonding between Dirac electrons in graphene and molecular charges at the interface. So, Dirac electrons at the interface between graphene and water are bonded to molecular charges and behave as quasiparticles with an effective mass that is about the mass of the water molecules. Future deeper theoretical analysis needs to be fulfilled to clarify the details of the voltage controlled Dirac cone reconstruction accompanied by electron bonding with molecular charges at the electrochemical graphene interface. Future studies of graphene obtained by CVD and exfoliation methods, bilayer and trilayer graphene, carbon nanotubes, other graphene-related systems, and other low-density-of-states systems in which quantum capacitance plays an important role are required to establish the applicability of the observed effects. Our findings are important for understanding the interfacial charging and discharging process and their applications in supercapacitors and selective chemical and biological sensors.

## Data Availability

The data presented in this study are available on request from the corresponding author.

## References

[1] Skrypnychuk V., Boulanger N., Nordenström A., Talyzin A. (2020). Aqueous activated graphene dispersions for deposition of high-surface area supercapacitor electrodes. J. Phys. Chem. Lett..

[2] Chen J., Li C., Shi G. (2013). Graphene materials for electrochemical capacitors. J. Phys. Chem. Lett..

[3] Butko A.V., Butko V.Y., Kumzerov Y.A. (2023). General capacitance upper limit and its manifestation for aqueous graphene interfaces. Int. J. Mol. Sci..

[4] Sadak O., Wang W., Guan J., Sundramoorthy A.K., Gunasekaran S. (2019). MnO_2_ nanoflowers deposited on graphene paper as electrode materials for Supercapacitors. ACS Appl. Nano Mater..

[5] Qorbani M., Esfandiar A., Mehdipour H., Chaigneau M., Irajizad A., Moshfegh A.Z. (2019). Shedding light on pseudocapacitive active edges of single-layer graphene nanoribbons as high-capacitance supercapacitors. ACS Appl. Energy Mater..

[6] Abdisattar A., Yeleuov M., Daulbayev C., Askaruly K., Tolynbekov A., Taurbekov A., Prikhodko N. (2022). Recent advances and challenges of current collectors for supercapacitors. Electrochem. Commun..

[7] Xiang C., Wu C.-W., Zhou W.-X., Xie G., Zhang G. (2021). Thermal transport in Lithium-Ion Battery: A micro perspective for thermal management. Front. Phys..

[8] Shen C., Li X., Li N., Xie K., Wang J., Liu X., Wei B. (2018). Graphene-boosted, high-performance aqueous Zn-Ion Battery. ACS Appl. Mater. Interfaces.

[9] von Wald Cresce A., Xu K. (2021). Aqueous lithium-Ion Batteries. Carbon Energy.

[10] Zhang Y., Xia X., Liu B., Deng S., Xie D., Liu Q., Wang Y., Wu J., Wang X., Tu J. (2019). Multiscale graphene-based materials for applications in sodium ion batteries. Adv. Energy Mater..

[11] Fedoseeva Y.V., Shlyakhova E.V., Vorfolomeeva A.A., Zaguzina A.A., Fedorenko A.D., Grebenkina M.A., Maksimovskii E.A., Shubin Y.V., Bulusheva L.G., Okotrub A.V. (2024). Iron-assisted synthesis of nitrogen-doped carbon material for sodium-ion batteries. J. Energy Storage.

[12] Ang P.K., Chen W., Wee A.T., Loh K.P. (2008). Solution-gated epitaxial graphene as ph sensor. J. Am. Chem. Soc..

[13] Zhang Y., de Aguiar H.B., Hynes J.T., Laage D. (2020). Water structure, dynamics, and sum-frequency generation spectra at electrified graphene interfaces. J. Phys. Chem. Lett..

[14] Green N.S., Norton M.L. (2015). Interactions of DNA with graphene and sensing applications of graphene field-effect transistor devices: A Review. Anal. Chim. Acta.

[15] Butko A.V., Butko V.Y., Lebedev S.P., Lebedev A.A., Davydov V.Y., Eliseyev I.A., Kumzerov Y.A. (2020). Detection of lysine molecular ions in solution gated field effect transistors based on unmodified graphene. J. Appl. Phys..

[16] Moradi R., Khalili N.P., Septiani N.L., Liu C., Doustkhah E., Yamauchi Y., Rotkin S.V. (2021). Nanoarchitectonics for abused-drug biosensors. Small.

[17] Tehrani Z., Burwell G., Azmi M.A., Castaing A., Rickman R., Almarashi J., Dunstan P., Beigi A.M., Doak S.H., Guy O.J. (2014). Generic epitaxial graphene biosensors for ultrasensitive detection of cancer risk biomarker. 2D Mater..

[18] Ding Y., Li C., Tian M., Wang J., Wang Z., Lin X., Liu G., Cui W., Qi X., Li S. (2023). Overcoming Debye length limitations: Three-dimensional wrinkled graphene field-effect transistor for ultra-sensitive adenosine triphosphate detection. Front. Phys..

[19] Sainz-Urruela C., Vera-López S., San Andrés M.P., Díez-Pascual A.M. (2021). Graphene-based sensors for the detection of bioactive compounds: A Review. Int. J. Mol. Sci..

[20] Li F., Huang Y., Huang K., Lin J., Huang P. (2020). Functional magnetic graphene composites for Biosensing. Int. J. Mol. Sci..

[21] Thangamuthu M., Hsieh K.Y., Kumar P.V., Chen G.-Y. (2019). Graphene- and graphene oxide-based nanocomposite platforms for electrochemical biosensing applications. Int. J. Mol. Sci..

[22] Kwak Y.H., Choi D.S., Kim Y.N., Kim H., Yoon D.H., Ahn S.-S., Yang J.-W., Yang W.S., Seo S. (2012). Flexible glucose sensor using CVD-grown graphene-based field effect transistor. Biosens. Bioelectron..

[23] Taniselass S., Arshad M.K.M., Gopinath S.C.B. (2019). Graphene-based electrochemical biosensors for Monitoring Noncommunicable Disease Biomarkers. Biosens. Bioelectron..

[24] Berninger T., Bliem C., Piccinini E., Azzaroni O., Knoll W. (2018). Cascading reaction of arginase and urease on a graphene-based FET for ultrasensitive, real-time detection of arginine. Biosens. Bioelectron..

[25] Wang Y., Li Y., Tang L., Lu J., Li J. (2009). Application of graphene-modified electrode for selective detection of dopamine. Electrochem. Commun..

[26] Rohaizad N., Sofer Z., Pumera M. (2020). Boron and nitrogen dopants in graphene have opposite effects on the electrochemical detection of explosive nitroaromatic compounds. Electrochem. Commun..

[27] Prabhakaran A., Nayak P. (2019). Surface Engineering of laser-scribed graphene sensor enables non-enzymatic glucose detection in human body fluids. ACS Appl. Nano Mater..

[28] Rabchinskii M.K., Sysoev V.V., Glukhova O.E., Brzhezinskaya M., Stolyarova D.Y., Varezhnikov A.S., Solomatin M.A., Barkov P.V., Kirilenko D.A., Pavlov S.I. (2022). Guiding graphene derivatization for the on-chip multisensor arrays: From the synthesis to the theoretical background. Adv. Mater. Technol..

[29] Rabchinskii M.K., Sysoev V.V., Varezhnikov A.S., Solomatin M.A., Struchkov N.S., Stolyarova D.Y., Ryzhkov S.A., Antonov G.A., Gabrelian V.S., Cherviakova P.D. (2023). Toward on-chip multisensor arrays for selective methanol and ethanol detection at room temperature: Capitalizing the graphene carbonylation. ACS Appl. Mater. Amp; Interfaces.

[30] Xia J., Chen F., Li J., Tao N. (2009). Measurement of the quantum capacitance of graphene. Nat. Nanotechnol..

[31] Du X., Guo H., Jin Y., Jin Q., Zhao J. (2015). Electrochemistry investigation on the graphene/electrolyte interface. Electroanalysis.

[32] Sharma P., Mišković Z.L. (2014). Capacitance of graphene in aqueous electrolytes: The effects of dielectric saturation of water and finite size of ions. Phys. Rev. B.

[33] Dankerl M., Hauf M.V., Lippert A., Hess L.H., Birner S., Sharp I.D., Mahmood A., Mallet P., Veuillen J., Stutzmann M. (2010). Graphene solution-gated field-effect transistor array for sensing applications. Adv. Funct. Mater..

[34] Chen S., Zhang Z.-B., Ma L., Ahlberg P., Gao X., Qiu Z., Wu D., Ren W., Cheng H.-M., Zhang S.-L. (2012). A graphene field-effect capacitor sensor in electrolyte. Appl. Phys. Lett..

[35] Heller I., Chatoor S., Männik J., Zevenbergen M.A., Dekker C., Lemay S.G. (2010). Influence of electrolyte composition on liquid-gated carbon nanotube and Graphene Transistors. J. Am. Chem. Soc..

[36] Yan F., Zhang M., Li J. (2013). Solution-gated graphene transistors for chemical and biological ‘sensors. Adv. Healthc. Mater..

[37] Luryi S. (1988). Quantum capacitance devices. Appl. Phys. Lett..

[38] Novoselov K.S., Geim A.K., Morozov S.V., Jiang D., Katsnelson M.I., Grigorieva I.V., Dubonos S.V., Firsov A.A. (2005). Two-dimensional gas of massless Dirac fermions in Graphene. Nature.

[39] Lopes L.C., Santos A., Bueno P.R. (2021). Measuring quantum conductance and capacitance of graphene using impedance-derived capacitance spectroscopy. Carbon.

[40] Hallock C.D., Rose M.J. (2024). Electrochemical impedance of well-passivated semiconductors reveals bandgaps, Fermi levels, and interfacial density of states. J. Am. Chem. Soc..

[41] Butko V.Y., Adams P.W. (2001). Quantum metallicity in a two-dimensional insulator. Nature.

[42] Butko V.Y., DiTusa J.F., Adams P.W. (2000). Coulomb gap: How a metal film becomes an insulator. Phys. Rev. Lett..

[43] Li G., Luican A., Andrei E.Y. (2009). Scanning tunneling spectroscopy of graphene on graphite. Phys. Rev. Lett..

[44] Butko A.V., Butko V.Y., Lebedev S.P., Lebedev A.A., Davydov V.Y., Smirnov A.N., Eliseyev I.A., Dunaevskiy M.S., Kumzerov Y.A. (2018). State memory in solution gated epitaxial graphene. Appl. Surf. Sci..

[45] Politano G.G., Vena C., Desiderio G., Versace C. (2020). Variable angle spectroscopic ellipsometry characterization of turbostratic CVD-grown bilayer and trilayer graphene. Opt. Mater..

[46] Li J., Pham P.H., Zhou W., Pham T.D., Burke P.J. (2018). Carbon-nanotube–electrolyte interface: Quantum and electric double layer capacitance. ACS Nano.

[47] Conway B.E. (2009). Electrochemical Supercapacitors: Scientific Fundamentals and Technological Applications.

